# An Investigation of Calibration Phantoms for CT Scanners with Tube Voltage Modulation

**DOI:** 10.1155/2013/563571

**Published:** 2013-12-25

**Authors:** Jing Zou, Xiaodong Hu, Hanyu Lv, Xiaotang Hu

**Affiliations:** State Key Laboratory of Precision Measuring Technology and Instruments, Tianjin University, Tianjin 300072, China

## Abstract

The effects of calibration phantoms on the correction results of the empirical artifacts correction method (ECCU) for the case of tube modulation were investigated. To improve the validity of the ECCU method, the effect of the geometry parameter of a typical single-material calibration phantom (water calibration phantom) on the ECCU algorithm was investigated. Dual-material calibration phantoms (such as water-bone calibration phantom), geometry arrangement, and the area-ratio of dual-material calibration phantoms were also studied. Preliminary results implied that, to assure the effectiveness of the ECCU algorithm, the polychromatic projections of calibration phantoms must cover the polychromatic projection data of the scanning object. However, the projection range of a water calibration phantom is limited by the scan field of view (SFOV), thus leading to methodological limitations. A dual-material phantom of a proper size and material can overcome the limitations of a single-material phantom and achieve good correction effects.

## 1. Introduction

In conventional computed tomography (CT) tube voltages are fixed (constant) while CT scans are carried out. However, tube voltage modulation is helpful for at least two reasons. First, tube voltage modulation has the potential for further dose reduction [1]. In addition, tube voltage modulation can obtain satisfactory image quality avoiding detector pixel saturation. When the thickness of the scanned object varies greatly with the view angle, the constant power of the X-ray generated by traditional CT may lead to pixel saturation or at least strike a balance of quantum noise among the different views. Dynamically adjusting the X-ray tube voltage in synchrony with the CT scanning can avoid this problem. Tube voltage modulation is very helpful in CT scanning, but when the voltage is modulated at different views during a CT scan, images reconstructed through commonly used reconstruction methods may contain various artifacts leading to inaccuracy and degradation of image quality.

Ideally, monochromatic X-ray beams are required in CT scanning. However, conventionally used X-ray beams in CT are polychromatic with a moderately broad energy spectrum. In this paper, different polychromatic X-ray beams are used through tube voltage modulating at different scanning views. As we know, X-ray attenuation processes in matter are energy dependent [2]. However, the reconstruction algorithm is based on the assumption of the monochromatic property of the X-ray beam that just computed the average attenuation coefficient and thus leads to the appearance of cupping artifacts or beam hardening artifacts within conventionally reconstructed images. Beam hardening effects in a fixed voltage are known to be one of the major sources of deterministic error. During recent decades, a number of correction methods have been developed, including physical approach, statistics approach, linearization, spectrum estimation, phantom calibration prereconstruction, threshold segmentation reprojection, and iteration method [3–9]. However, artifact correction in the case of tube modulation during a CT scan has been primarily addressed in the literature. “Water calibration for CT scanners with tube voltage modulation” in 2010, in which the ECCU algorithm for cupping artifact correction was provided, showed excellent simulation results. The typical artifact correction applied in clinical CT scanners is known as water correction, and so water phantom is used in the ECCU method [10]. To ensure the integrity of the algorithm, large water phantoms are required for routine calibrations. However, the size of the calibration phantom is limited by the scan field of view (SFOV), which may lead to methodological limitations.

In this paper, the effects of calibration phantoms for CT scanners with tube voltage modulation are investigated by a numerical simulation method. In Section 2, we provide a brief review of the ECCU algorithm, followed by a numerical implementation of the ECCU algorithm. In Section 3, we have performed two sets of simulation experiments to investigate the effect of the geometry parameter of water phantoms on the ECCU algorithm. Then, we investigate the effect of dual-material calibration phantoms on the ECCU algorithm, including the area-ratio and geometrical arrangement of different materials. Finally, conclusions and remarks are given in Section 4.

## 2. Principle of the ECCU Method

The log attenuation of a polychromatic X-ray spectrum, as used for CT, is given as

(1) $$\begin{array}{c} q\left(U,L\right)=-\ln\omega \left(U,L,E\right)e^{-\int \mu (E,r)dL}dE, \end{array}$$

where *L* is the line of integration corresponding to the ray direction, **r** represents the position vector, *μ*(*E*, **r**) indicates the energy-dependent spatial distribution of the linear attenuation coefficient, *E* is the photon energy, and *ω*(*U*, *L*, *E*) is the normalized spectrum distribution of the emitted X-rays at tube voltage *U*.

Assuming the decomposition *μ*(*E*, **r**) = *φ*(*E*)*f*(**r**), we will get the following formula:

(2) $$\begin{array}{c} q\left(U,p\right)=-\ln\int \omega \left(U,L,E\right)e^{p\varphi (E)}dE\;\text{with}\;\;p=\int f\left(r\right)dL, \end{array}$$

where *φ*(*E*) is the energy dependence of the most prominent material in the object.

Let *q* be the polychromatic projection data and *p* = *D*(*U*, *q*) the desired monochromatic material-specific projection data. The aim of the ECCU algorithm is to acquire proper *p* from *q*(*U*, *p*). Let *p* = *D*(*U*, *q*), which is some yet unknown decomposition function, as follows:

(3) $$\begin{array}{c} D\left(U,q\right)=\overset{N-1}{\underset{n=0}{\sum }}c_{n}b_{n}\left(U,q\right)=c\cdot b\left(U,q\right), \end{array}$$

where *D*(*U*, *q*) represents a linear combination of basis functions *b*_*n*_(*U*, *q*) and *b*_*n*_(*U*, *q*) = *U* ^*l*^ *q* ^*k*^ as basis functions with *k* = 0,…, *K* and *l* = 0,…, *L*. A set of (*K* + 1)(*L* + 1) basis images are defined as *f*_*n*_(**r**) = *R* ^−1^ *b*_*n*_(*U*, *q*), *R* ^−1^ represents the inverse radon transform, and *f*_*n*_ is the reconstruction image of the projection data *q* after they have been passed through the basis functions *b*_*n*_(*U*, *q*).

Now, the reconstruction of the material-selective projection data can be written as a linear combination of these basis images:

(4) f(r)=R−1D(U,q)=R−1∑cnbn(U,q)=∑cnR−1bn(U,q)=cnfn(r)=c·f(r).

The set of coefficients **c** is solved through minimizing the least-squares deviation:

(5) $$\begin{array}{c} E^{2}=\int {w\left(r\right)\left(cf\left(r\right)-t\left(r\right)\right)}^{2}d^{2}r, \end{array}$$

where *t*(**r**) represents a given template image and *w*(**r**) is the weight image used to suppress the contribution of unwanted structures of the calibration object.

The process of the ECCU method is calculating the coefficients **c** through calibration phantom. Furthermore, these coefficients are used to preprocess projection data acquired from ordinary scanning through formula (3). Concrete details have been shown in the literature [10]. Influence of calibration phantom material and calibration phantom size on the ECCU method is investigated in the following section.

## 3. Calibration Phantom Study

In this section, the effect of different calibration phantoms including single-material phantom and dual-material phantom on the ECCU method is investigated by using computer simulation data. A projection simulation based on a physical imaging model and scanning phantom simulation is introduced in Section 3.1. The investigation of single-material calibration phantom and dual-material calibration phantom is implemented in Sections 3.2 and 3.3.

### 3.1. Simulations

To simulate the projection data of different phantoms acquired with a polychromatic X-ray source, we utilize the same scheme as in [10]. The distribution function of a polychromatic X-ray energy spectrum emitted by X-ray tube GE Maxi Ray 125 [spectrum  GUI] and the absorption attenuation coefficients were interpolated from the database of NIST (National Institute of Standards and Technology's website) [11]. We first computed the linear integral projection of every material that formed the phantom and then computed corresponding polychromatic projections using a different spectrum.

**Figure 1:**
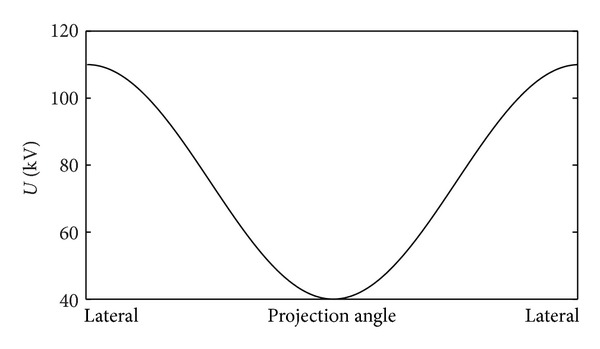
Tube voltage varied sinusoidally from 110 kV to 40 kV.

**Figure 2:**
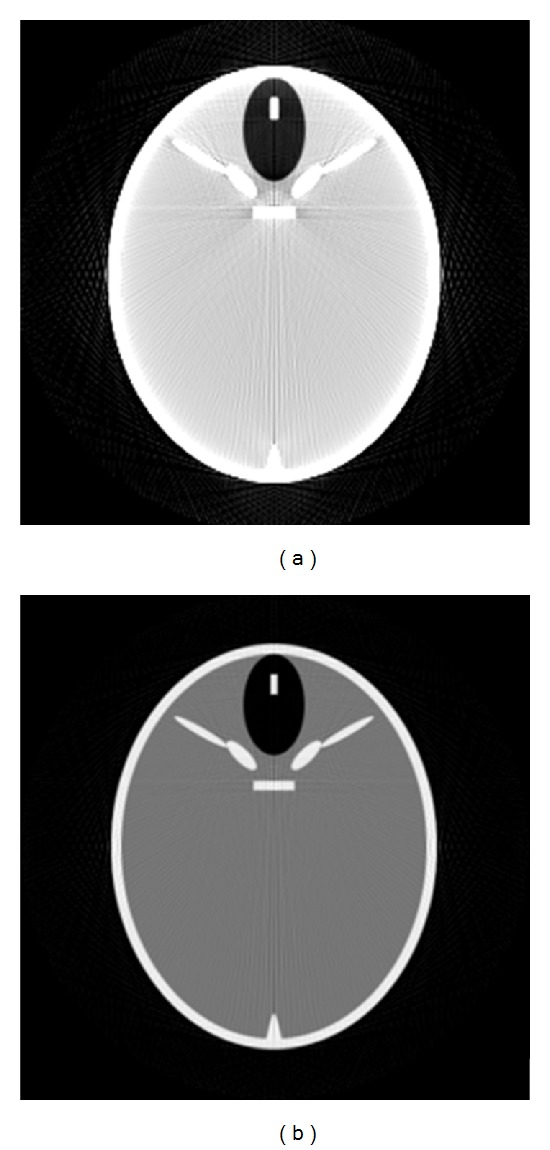
Reconstruction images of FORBILD head phantom. (a) Directly reconstructed image from tube voltage modulation projection. (b) Reconstructed image from monochromatic projection data.

To investigate the effect of calibration phantoms on the ECCU algorithm, the well-known FORBILD head phantom as a test phantom was simulated. More detailed information about the phantom is listed in Table 1. The geometry parameters of the virtual CT system for scanning FORBILD head phantom are as follows. The distance from source to rotation center is 570 cm and the distance from source to detector is 435 cm. Detector array is 256 × 256 with a pixel size of 0.2067 cm. The tube voltage *U* as a function of the projection angle *α* is shown in Figure 1. Subsequently, we simulated projection data of FORBILD head phantom using voltage modulation curves shown in Figure 1, corresponding directly to the reconstructed image shown in Figure 2(a). In Figure 2(b), we show a reconstructed image from the monochromatic projection data as a contrast. In addition, all simulations were carried out using the voltage modulation profile in Figure 1.

### 3.2. Single-Material Calibration Phantom

**Figure 3:**
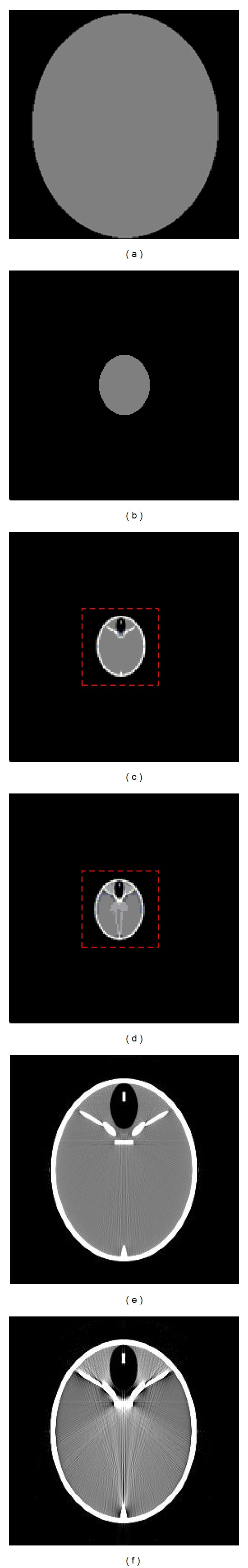
Water calibration phantoms and corresponding corrected images of FORBILD head phantom. (a) Elliptical cylinder phantom with semiaxes 48 cm and 40 cm. (b) Elliptical cylinder phantom with semiaxes 28 cm and 12 cm. (c) Corrected image using calibration phantom shown in (a). (d) Corrected image using calibration phantom shown in (b). (e) and (f) are zoomed-in images of the area enclosed by the red squares in (c) and (d). Display window is [0,600] HU.

In order to understand the effect of object size on the ECCU method, a series of virtual solid water phantoms with different geometry sizes were investigated. Here, two water phantoms are given as examples to illustrate the effect of geometry size on the ECCU method. The first elliptical cylinder water phantom with semiaxes 48 cm and 40 cm is shown in Figure 3(a). The second with semiaxes 28 cm and 12 cm is shown in Figure 3(b). Corresponding correction coefficients of the two calibration phantoms are computed through the ECCU method. Then, the correction coefficients are used to correct the tube voltage modulated projection of the FORBILD head phantom. The correction results through phantom Figures 3(a) and 3(b) are shown in Figures 3(c) and 3(d). To show images of a proper ratio, the SFOV of Figures 3(c) and 3(d) are simply reduced to 1/3, which is enclosed by a red square, and the corresponding zoomed images are shown in Figures 3(e) and 3(f). From Figure 3(e), we can see that the corrected image is as good as the reconstructed image from the monochromatic projection shown in Figure 2(b), which demonstrates that the ECCU method is effective when the large water phantom is used. However, severe errors appear in the corrected images when the small water calibration phantom is used, as shown in Figure 3(f). In order to analyze the reason leading to the failure of the ECCU method, we investigated the maximum and minimum values of tube voltage modulated projection data, including water phantom in Figures 3(a) and 3(b) and FORBILD head phantom in Figure 2(a). The corresponding results are shown in Table 2. Although the size of the calibration phantom in Figure 3(b) is much the same as that of the FORBILD head phantom, we can see that the scope of the polychromatic projection data of water phantom in Figure 3(b) cannot cover the polychromatic projection data of the FORBILD head phantom in Figure 2(a), which may be the key reason leading to the failure of the ECCU method.

**Figure 4:**
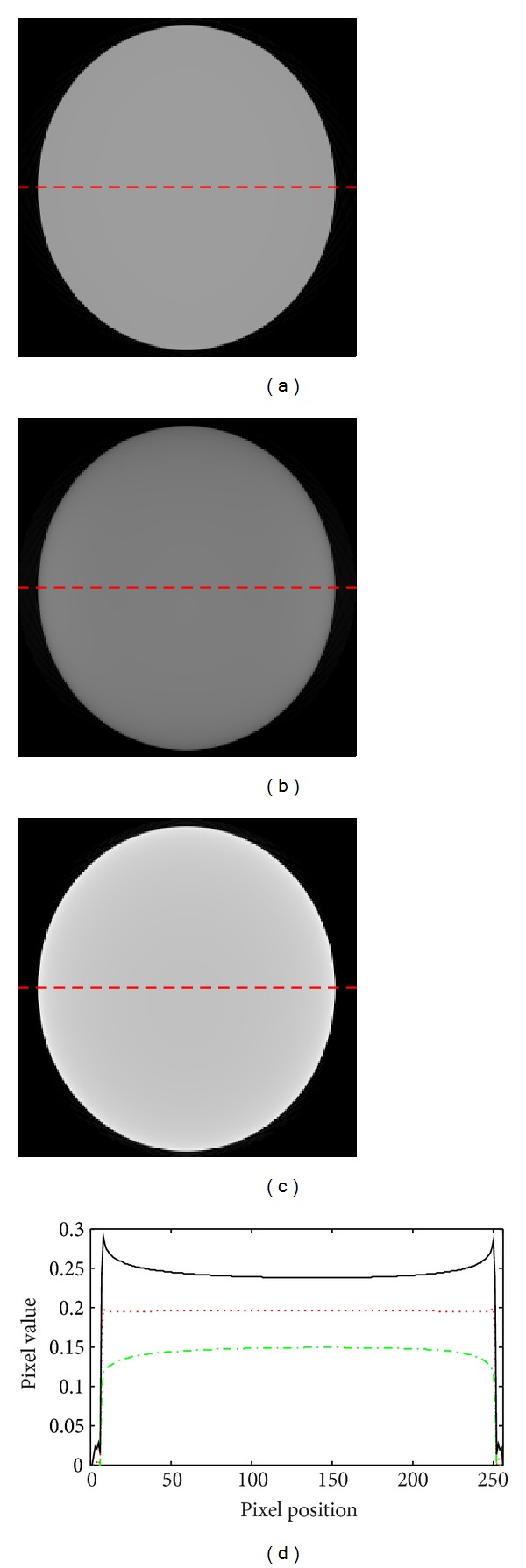
Images of water phantom. (a) Corrected image using water calibration phantom shown in Figure 3(a). (b) Corrected image using water calibration phantom shown in Figure 3(b). (c) Reconstructed image without correction. (d) Intensity plots for lines through the reconstructed images of (a), (b), and (c): the dot line, the dash-dot line, and the solid line represent intensity profiles of the central horizontal line in (a), (b), and (c), respectively.

To verify that the above analysis is reasonable, a test phantom made of water was investigated. The semiaxes of a water elliptical cylinder are 24 cm and 22 cm. From the geometry size of Figures 3(a) and 3(b), we know that the size of Figure 3(a) is larger than that of the actual testing phantom, but Figure 3(b) is smaller than the actual testing phantom. Again, correction coefficients of water calibration phantom in Figures 3(a) and 3(b) are used to implement the ECCU method. Corrected images using calibration phantoms in Figures 3(a) and 3(b) are shown in Figures 4(a) and 4(b). The directly reconstructed image using tube voltage modulated projection data is shown in Figure 4(c) and cupping artifacts appeared. In order to quantify the accuracy of the three reconstructed images, the profiles along Figures 4(a), 4(b), and 4(c) are displayed in Figure 4(d). From these profiles, it can be observed that the profile of Figure 4(a) is a straight line, but the profiles of Figures 4(b) and 4(c) are curves, which demonstrates that when the scanning phantom to be corrected is smaller than the standard calibration phantom, cupping artifacts (see the solid line in Figure 4(d)) disappear. However, when the scanning phantom to be corrected is larger than the calibration phantom, the correction method is invalid (see the dash-dot line in Figure 4(d)). It should be reasonable to assume that the ECCU method required that the geometry of the calibration phantom be larger than that of the testing phantom when they are made of the same material. Actually, the maximum size of the calibration phantom is limited due to the actual SFOV; therefore the material and geometry design of the calibration phantom are of great importance.

### 3.3. Dual-Material Calibration Phantom

Actually, the maximum SFOV in the CT system is designed according to the specified parameters of the scanning objects. With high-density objects that occupy the maximum SFOV, an identically sized water calibration phantom may not be appropriate for the implementation of the ECCU method, because the polychromatic projection data of the water phantom cannot cover that of the scanning object. Inserting a high-density mass material in the water phantom is a direct way to solve this problem. Based on this idea, dual-material calibration phantoms are adopted and studied in this section. We have to mention that the polychromatic projections of dual-material calibration phantoms in this section could cover the polychromatic projection of the FORBILD head phantom, which are ensured by properly adjusting the ratio of the two materials in the fixed SFOV.

**Figure 5:**
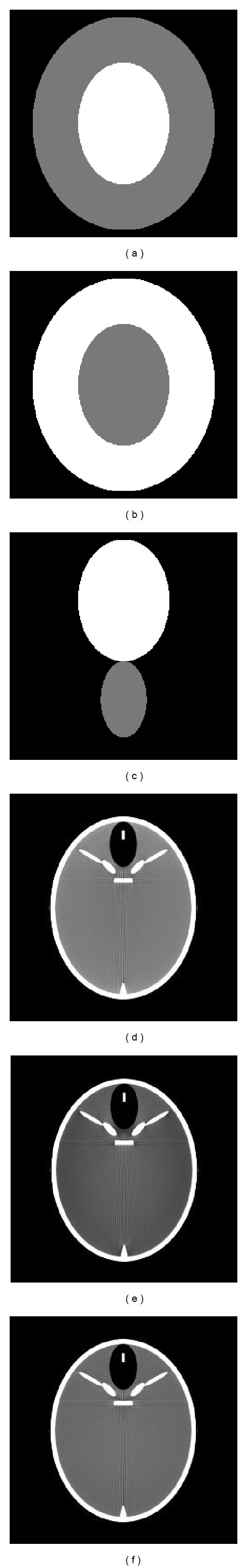
Dual-material calibration phantoms and corresponding correction images of FORBILD head phantom. The first row shows dual-material calibration phantom with different sizes. (a) A 14 × 12 cm oval water phantom with 8 × 6 cm bone insert. (b) A 14 × 12 cm oval bone phantom with 8 × 6 cm water insert. (c) A two-cylinder phantom; the upper one is an 8 × 6 cm bone cylinder and the lower one is a 5 × 3 cm water elliptical cylinder. (d), (e), and (f) are correction images of FORBILD head phantom using calibration phantoms shown in (a), (b), and (c), respectively.

Firstly, the effect of the geometrical arrangement of the dual-material phantom on the ECCU method was investigated. We conducted two cylinder-in-cylinder phantoms: one cylinder-in-cylinder phantom was a 14 × 12 cm oval water phantom with an 8 × 6 cm bone insert, as shown in Figure 5(a). The other was a 14 × 12 cm oval bone phantom with an 8 × 6 cm water insert, as shown in Figure 5(b). In addition, a two-cylinder phantom was simulated, as shown in Figure 5(c); the upper one was an 8 × 6 cm bone cylinder and the lower one was a 5 × 3 cm water cylinder. Again, corresponding correction coefficients of the three calibration phantoms were computed through the ECCU method. These correction coefficients are then used to correct the tube voltage modulated projection of the FORBILD head phantom. The correction results through phantom Figures 5(a), 5(b), and 5(c) are shown in Figures 5(d), 5(e), and 5(f), respectively. Compared with the directly reconstructed image in Figure 2(a), the corrected images in Figures 5(d), 5(e), and 5(f) are almost free of artifacts, although the uniformity of Figure 5(e) is not very good.

**Figure 6:**
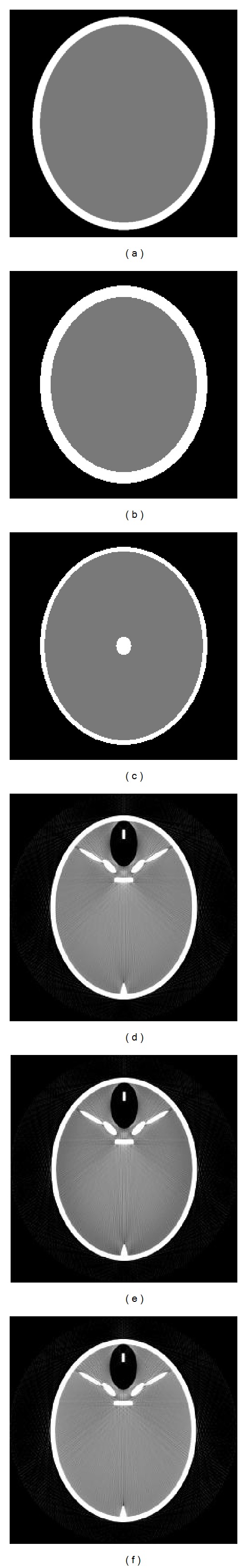
Dual-material calibration phantoms and corresponding correction images of FORBILD head phantom. The first row shows dual-material calibration phantom with different sizes. (a) A 14 × 12 cm oval bone phantom with 13 × 11 cm water insert. (b) A 13 × 11 cm oval bone phantom with 11.5 × 9.6 cm water insert. (c) A 13 × 11 cm oval bone phantom with 12.4 × 10.4 cm water insert, in which a 1.2 × 1.0 cm oval bone is inserted in the center. (d), (e), and (f) are correction images of the FORBILD head phantom using calibration phantoms shown in (a), (b), and (c), respectively.

For a more detailed analysis on the influence of cylinder-in-cylinder phantoms similar to Figure 5(b) on the ECCU method, three cylinder-in-cylinder phantoms were investigated. The first one was a 14 × 12 cm oval bone phantom with a 13 × 11 cm water insert, as shown in Figure 6(a). The second one was a 13 × 11 cm oval bone phantom with a 11.5 × 9.6 cm water insert, as shown in Figure 6(b) and the last one was 13 × 11 cm oval bone phantom with a 12.4 × 10.4 cm water insert, in which a 1.2 × 1.0 cm oval bone was inserted in the center, as shown in Figure 6(c). Corresponding corrected images of the FORBILD head phantom through phantom Figures 6(a), 6(b), and 6(c) are shown in Figures 6(d), 6(e), and 6(f). We can see that bright artifacts appear in the area surrounded by the high-density materials; also streak artifacts can be observed among the higher density materials. This phenomenon shows that the geometrical arrangement of Figures 6(a), 6(b), and 6(c) is not good enough compared with calibration phantoms such as Figures 5(a) and 5(c) even though the contrast of the FORBILD image is improved to a certain degree.

**Figure 7:**
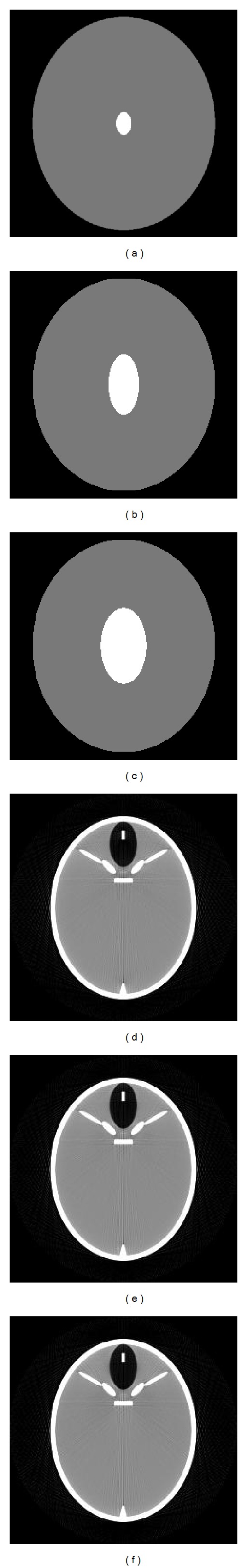
Dual-material calibration phantoms and corresponding correction images of FORBILD head phantom. The first row shows dual-material calibration phantom with different sizes. (a) A 14 × 12 cm oval water phantom with a 1.5 × 1.0 cm bone insert. (b) A 14 × 12 cm oval water phantom with 4 × 2 cm bone insert. (c) A 14 × 12 cm oval water phantom with 5 × 3 cm bone insert. (d), (e), and (f) are correction images of FORBILD head phantom using calibration phantoms shown in (a), (b), and (c).

Lastly, the effect of the area-ratio of the dual-material phantom on the ECCU method was investigated through cylinder-in-cylinder phantoms as in Figure 5(a). The first cylinder-in-cylinder phantom was a 14 × 12 cm oval water phantom with a 1.5 × 1.0 cm bone insert, as shown in Figure 7(a). The second one was a 14 × 12 cm oval water phantom with a 4 × 2 cm bone insert, as shown in Figure 7(b). The last one was a 14 × 12 cm oval water phantom with a 5 × 3 cm bone insert, as shown in Figure 7(c). The area-ratio of outer water phantom to inserted bone phantom for Figures 7(a), 7(b), and 7(c) is 111 : 1, 20 : 1, and 10.2 : 1, respectively. The corresponding projection ranges of these phantoms are shown in Table 3, which show that the polychromatic projections of the calibration phantom can cover the polychromatic projection data of the scanning object. Again, the ECCU method is implemented through these calibration phantoms, and the corresponding corrected images of the FORBILD head phantom through calibration phantom Figures 7(a), 7(b), and 7(c) are shown in Figures 7(d), 7(e), and 7(f). It can be observed that the corrected images agree well with the reconstructed image from the monochromatic projection data shown in Figure 2(b), which demonstrates that this kind of cylinder-in-cylinder phantom is valid for the ECCU method. Also, the area-ratio may not influence the validity of the ECCU method in case of this kind of cylinder-in-cylinder phantom.

## 4. Conclusions

In this paper, calibration phantoms for CT scanners with tube voltage modulation are investigated through computer simulation. We investigated the effect of the geometry parameter of the water calibration phantom on the ECCU algorithm. The simulation results show that the large water phantom can improve image quality, whilst a small water phantom may degrade image quality, because the polychromatic projection of the water phantom cannot cover the polychromatic projection of the scanning object. We find that in order to assure the effectiveness of the ECCU algorithm, the polychromatic projections of the calibration phantom must cover the polychromatic projection data of the scanning object. To assure the validity of the ECCU method, dual-material calibration phantoms are introduced. Furthermore, the geometry arrangement and the area-ratio of dual-material calibration phantoms were investigated. According to the numerical results, it can be concluded that dual-material calibration phantoms are valid in removing “cupping artifacts” and “streak artifacts”.

## Figures and Tables

**Table 1:** Parameters of FORBILD head phantom.

Label	Geometry	Position (cm)	Ellipsoid	Elliptical cylinder/cone	Φ (°)	*θ* (°)
			half-axis length (cm, cm, cm)	(radius, radius, length) (cm, cm, cm)		
1	Ellipsoid	(0, 0, 0)	(9.6, 12.0, 12.5)	N/A	0.0	0.0
2	Ellipsoid	(0, 0, 0)	(9, 11.4, 11.9)	N/A	0.0	0.0
3	Ellipsoid	(0, 8.4, 0)	(1.8, 3.0, 3.0)	N/A	0.0	0.0
4	Elliptical cylinder	(0, 3.6, 0)	N/A	(4, 1.2, 0.483)	60	90
5	Elliptical cylinder	(0, 9.6, 0)	N/A	(2, 0.525661, 0.4)	−90	−30
6	Ellipsoid	(−1.9, 5.4, 0)	(1.165, 0.406, 3)	N/A	0.0	−45
7	Ellipsoid	(1.9, 5.4, 0)	(1.165, 0.406, 3)	N/A	0.0	45
8	Elliptical cylinder	(−4.3, 6.8, −1)	N/A	(1.8, 0.24, 4)	0.0	−30
9	Elliptical cylinder	(4.3, 6.8, −1)	N/A	(1.8, 0.24, 4)	0.0	30
10	Cone	(0, −10.15, −0.2)	N/A	(0.5, 0.2, 1.5)	0.0	0.0

**Table 2:** Scope of projection data for different phantoms.

Phantom	Scope of projection data
Figure 2(a)	[0,12.7046]
Figure 3(a)	[0,29.9094]
Figure 3(b)	[0,9.11191]

**Table 3:** Scope of projection data for different phantoms.

Phantom	Scope of projection data
Figure 2(a)	[0,12.7046]
Figure 7(a)	[0,12.9765]
Figure 7(b)	[0, 17.6509]
Figure 7(c)	[0,19.4019]
